# Supplementary material for: Prediction of LncRNA-encoded small peptides in glioma and oligomer channel functional analysis using in silico approaches
Source: PLoS One. 2021 Mar 18;16(3):e0248634. doi: 10.1371/journal.pone.0248634 (PMC7971536; doi:10.1371/journal.pone.0248634)
Supplement: S1 Fig — The red and green dots represent upregulate and downregulated RNAs respectively. (DOCX) [file pone.0248634.s001.docx]

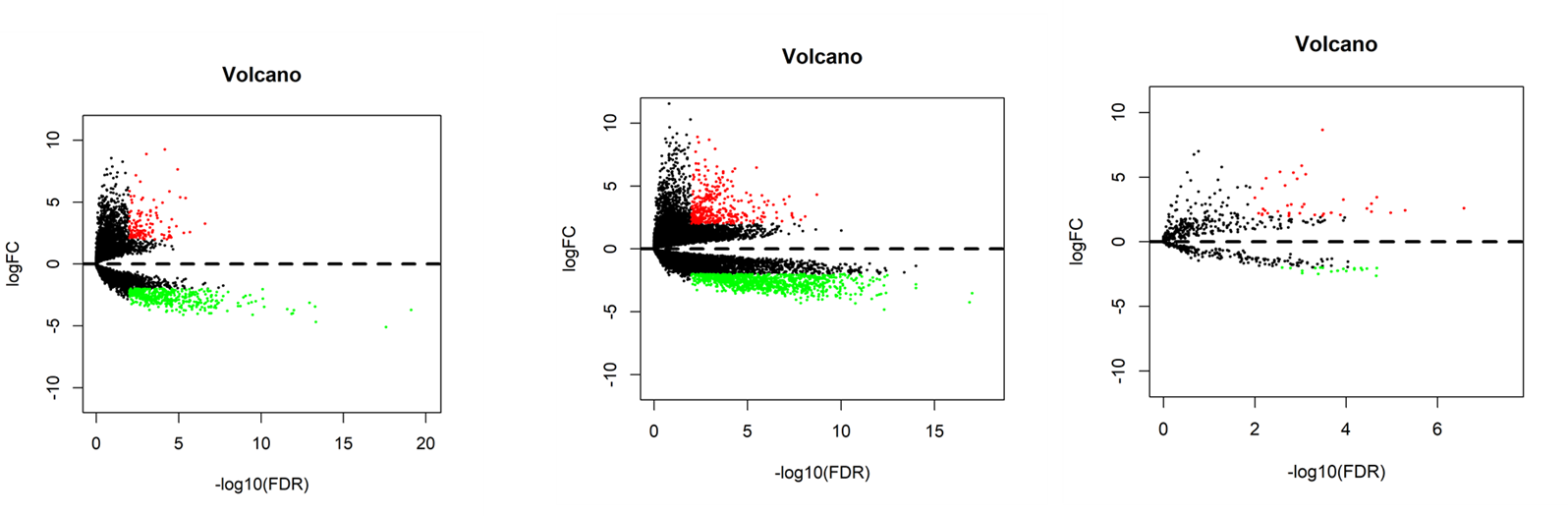


**lncRNA mRNA miRNA**

S1 Fig. The volcano of lncRNA, mRNA and miRNA. The red and green dots represent upregulate and downregulated RNAs respectively.
