# Supplementary material for: Prediction of LncRNA-encoded small peptides in glioma and oligomer channel functional analysis using in silico approaches
Source: PLoS One. 2021 Mar 18;16(3):e0248634. doi: 10.1371/journal.pone.0248634 (PMC7971536; doi:10.1371/journal.pone.0248634)
Supplement: S2 Fig — The DLEU1 contain ORF1 and ORF8 can encoding small peptides. (DOCX) [file pone.0248634.s002.docx]

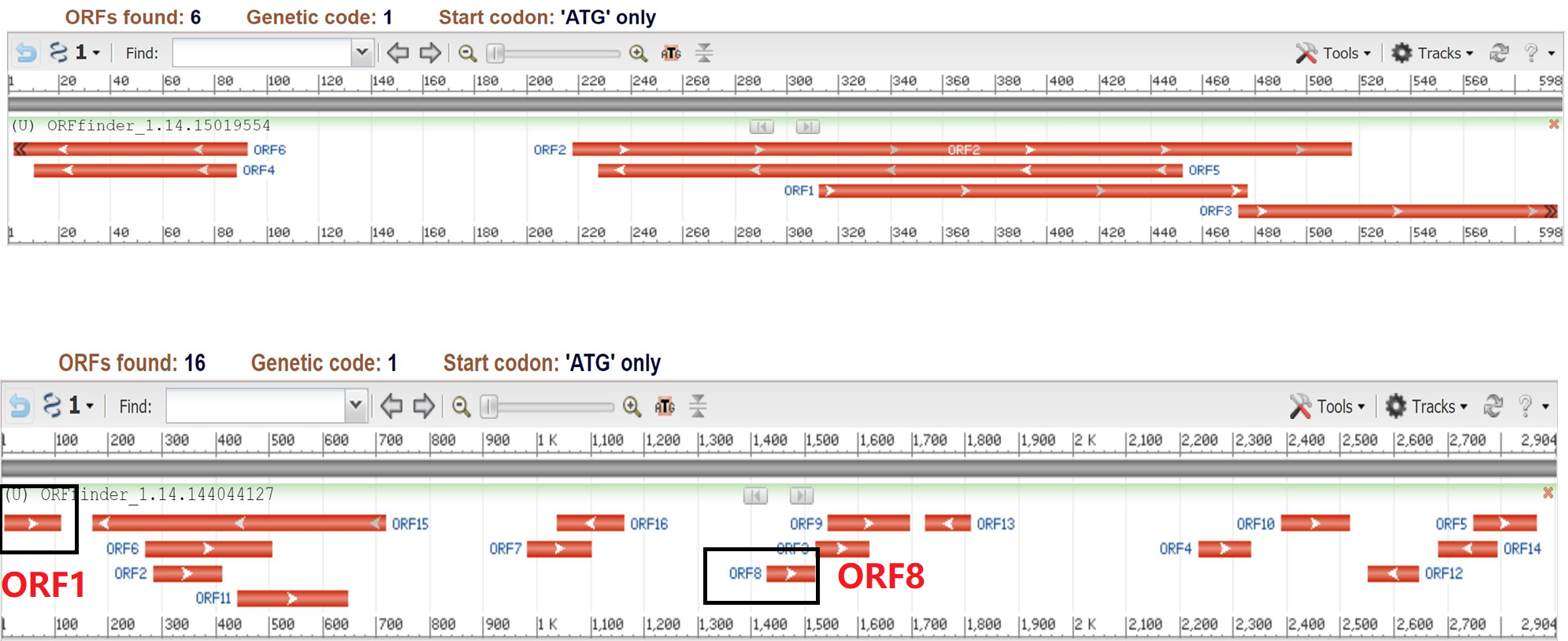


S2 Fig The predicted ORFs in WARS2.IT1 and DLEU1 lncRNA . the DLEU1 contain ORF1 and ORF8 can encoding small peptides.
