# Supplementary material for: Prediction of LncRNA-encoded small peptides in glioma and oligomer channel functional analysis using in silico approaches
Source: PLoS One. 2021 Mar 18;16(3):e0248634. doi: 10.1371/journal.pone.0248634 (PMC7971536; doi:10.1371/journal.pone.0248634)
Supplement: S3 Fig — (DOCX) [file pone.0248634.s003.docx]

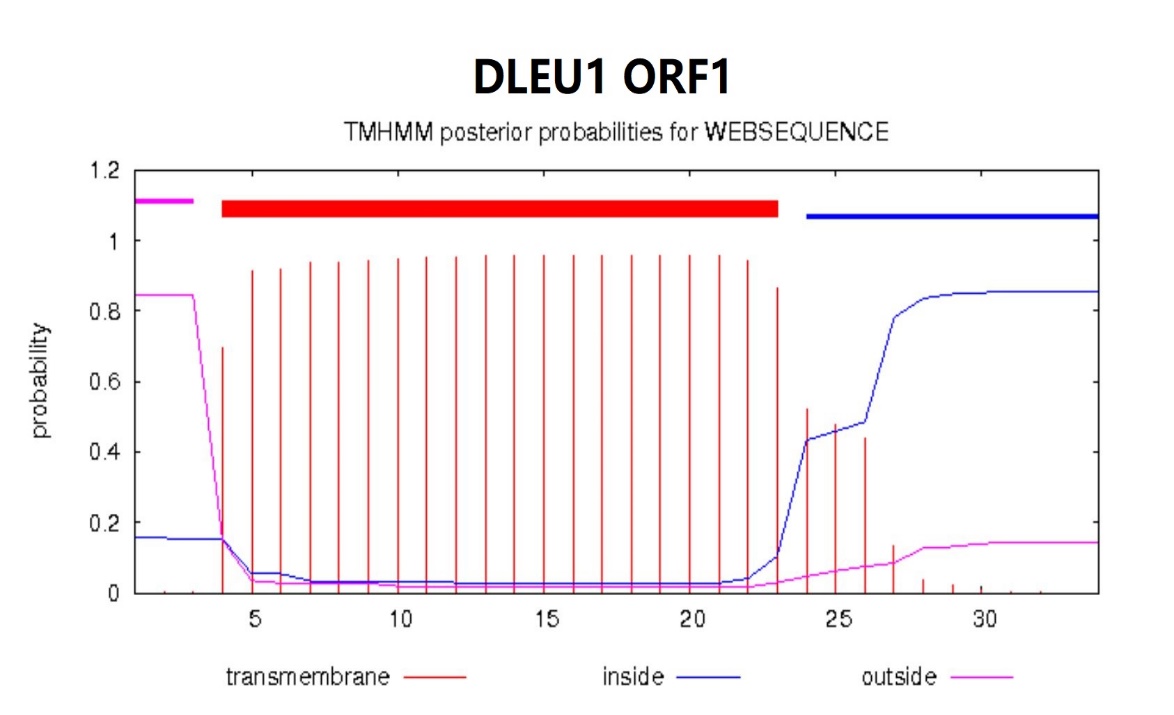

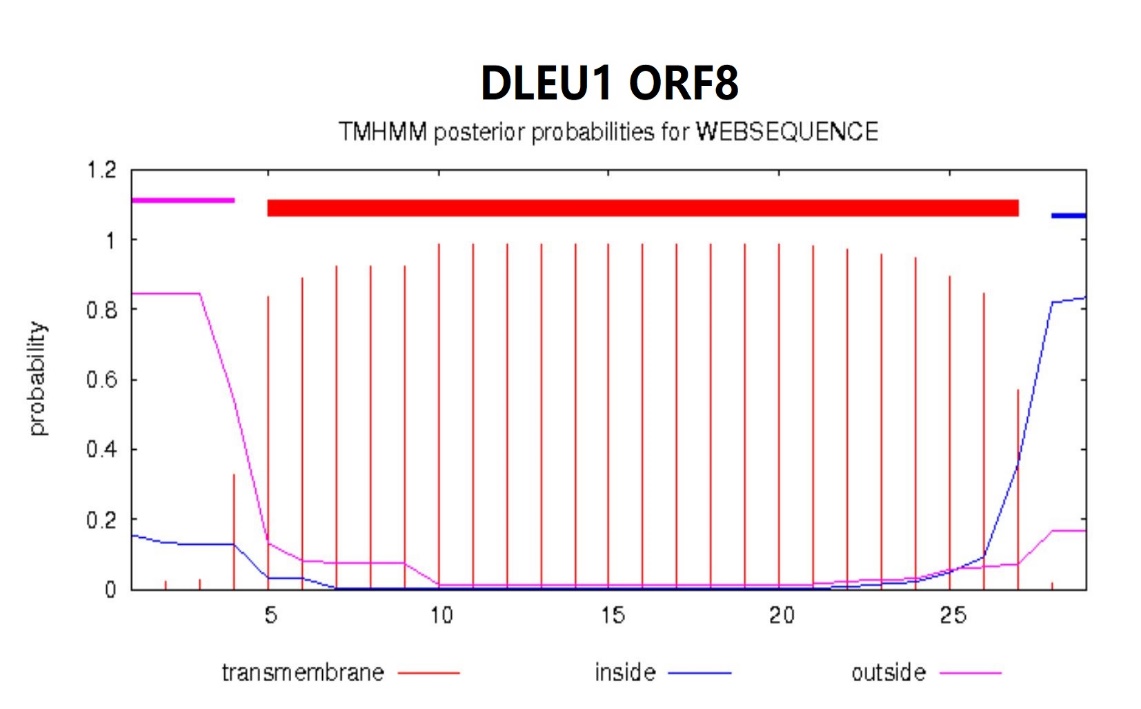


S3 Fig, Two ORFs second structure, the red lines represent a predicted transmembrane α-helix configuration.
