# Supplementary figures and images for: Prediction of LncRNA-encoded small peptides in glioma and oligomer channel functional analysis using in silico approaches
Source: PLoS One. 2021 Mar 18;16(3):e0248634. doi: 10.1371/journal.pone.0248634 (PMC7971536; doi:10.1371/journal.pone.0248634)

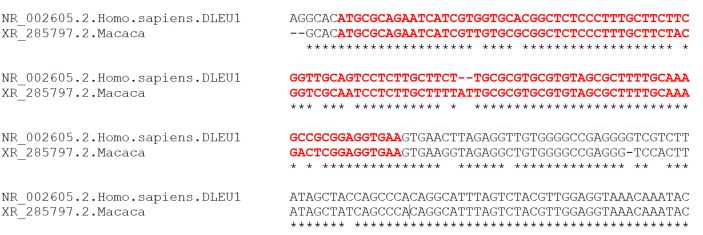


DLEU1 ORF1


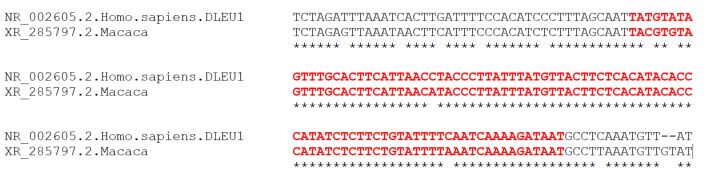


DLEU1 ORF8

S4 Fig, Comparison of Two ORFs sequence conservation of DLEU1.

DLEU1 ORF8

Supplement: S4 Fig — (DOCX) [file pone.0248634.s004.docx]

The 11 hub lncRNA’s Kaplan–Meier survival curves


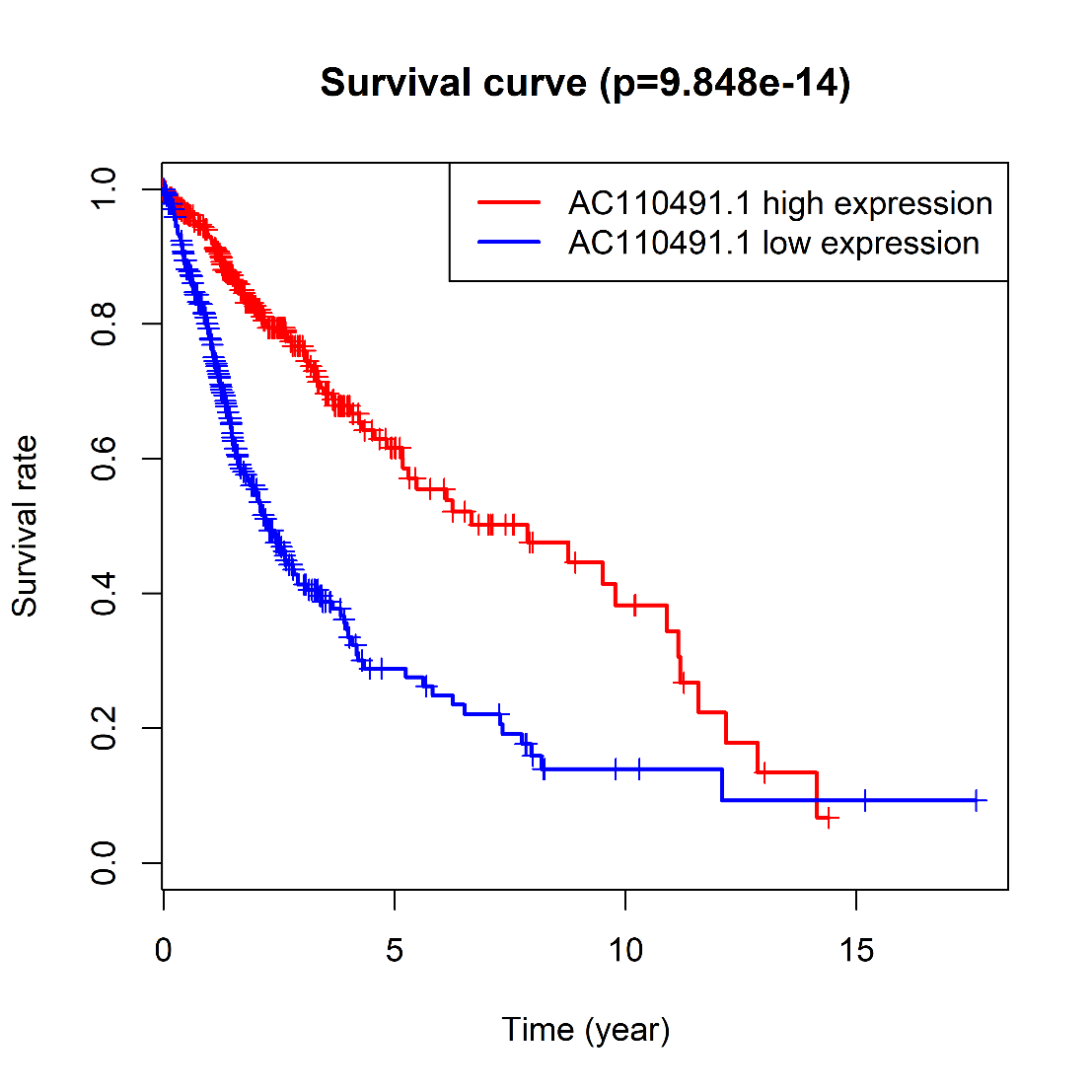

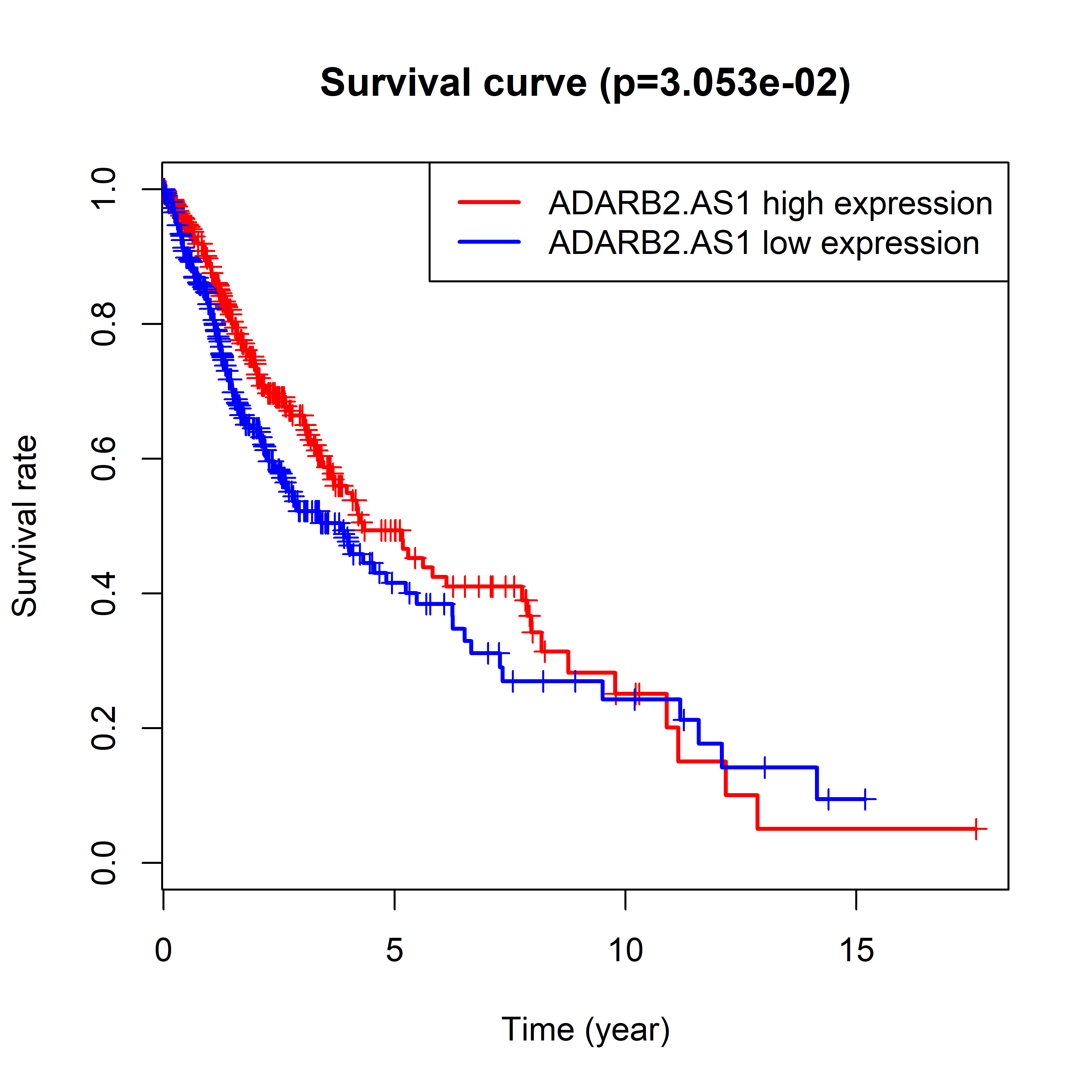

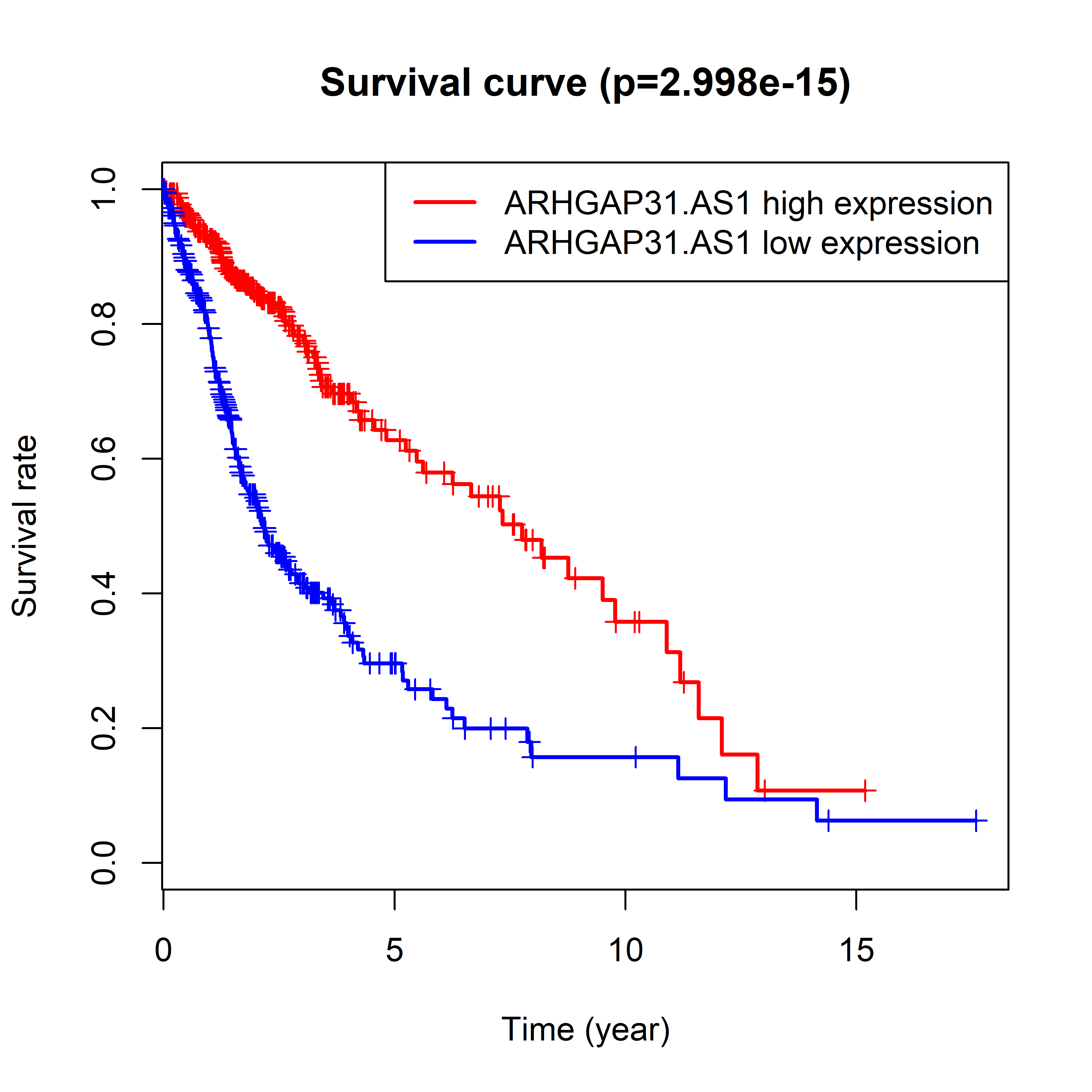

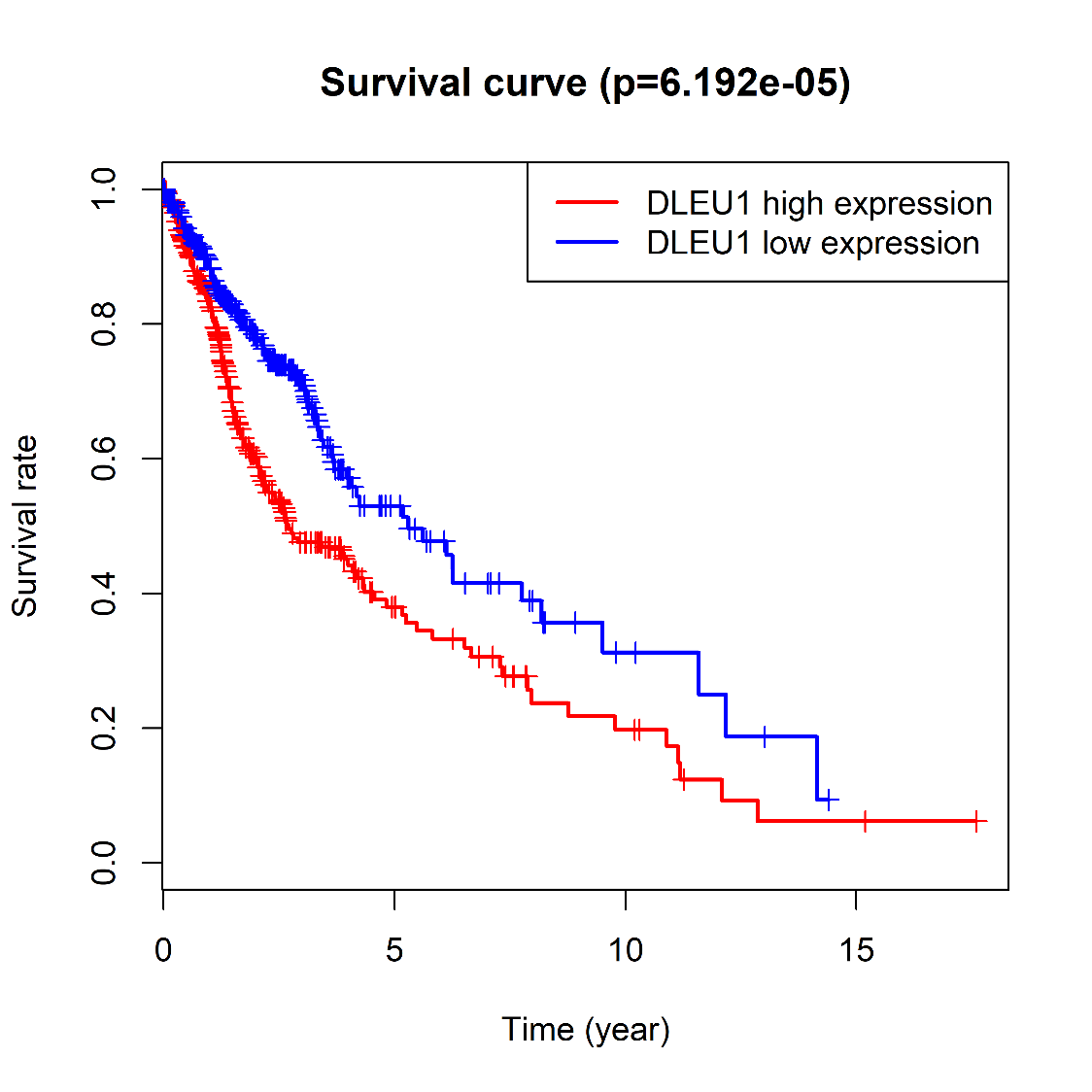

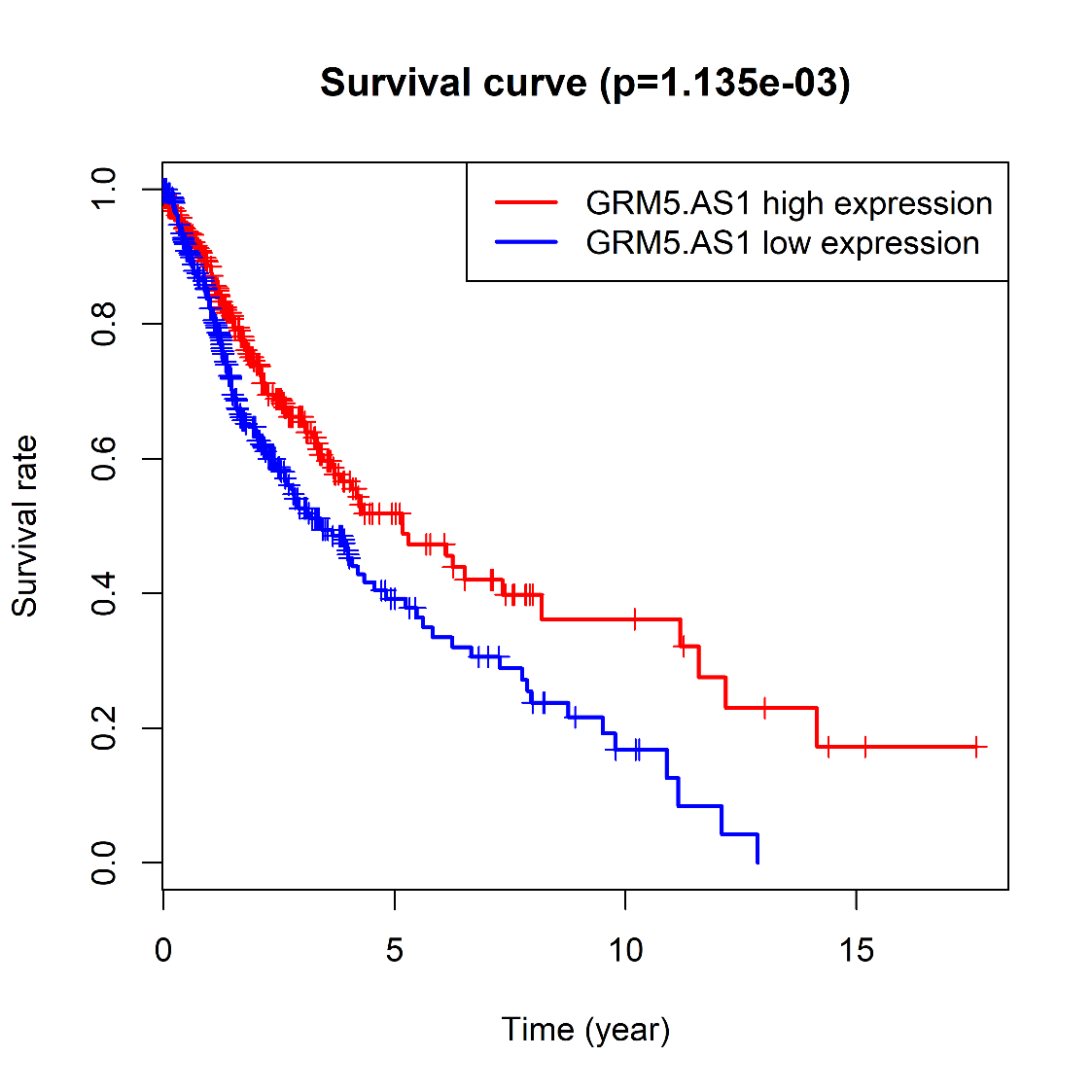

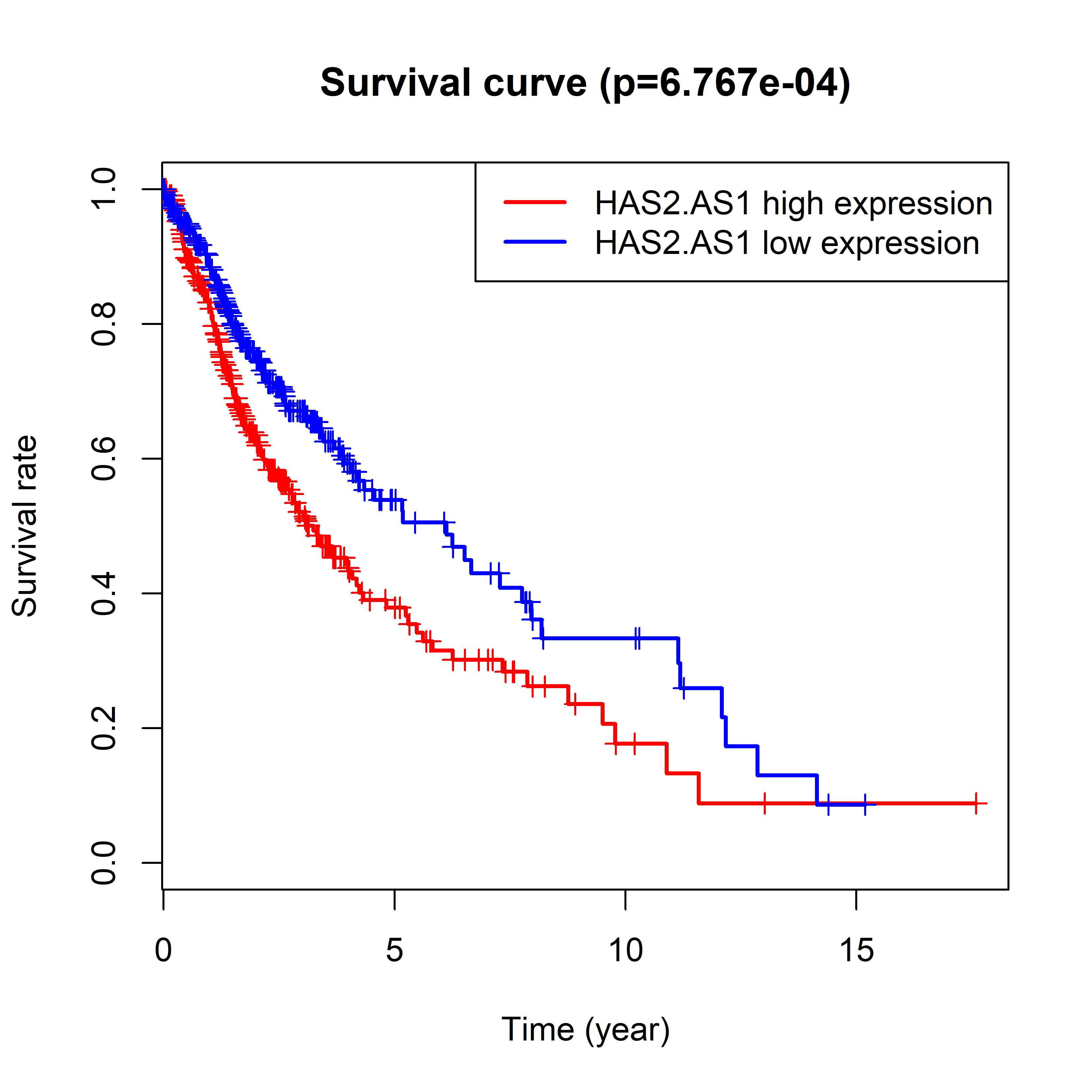

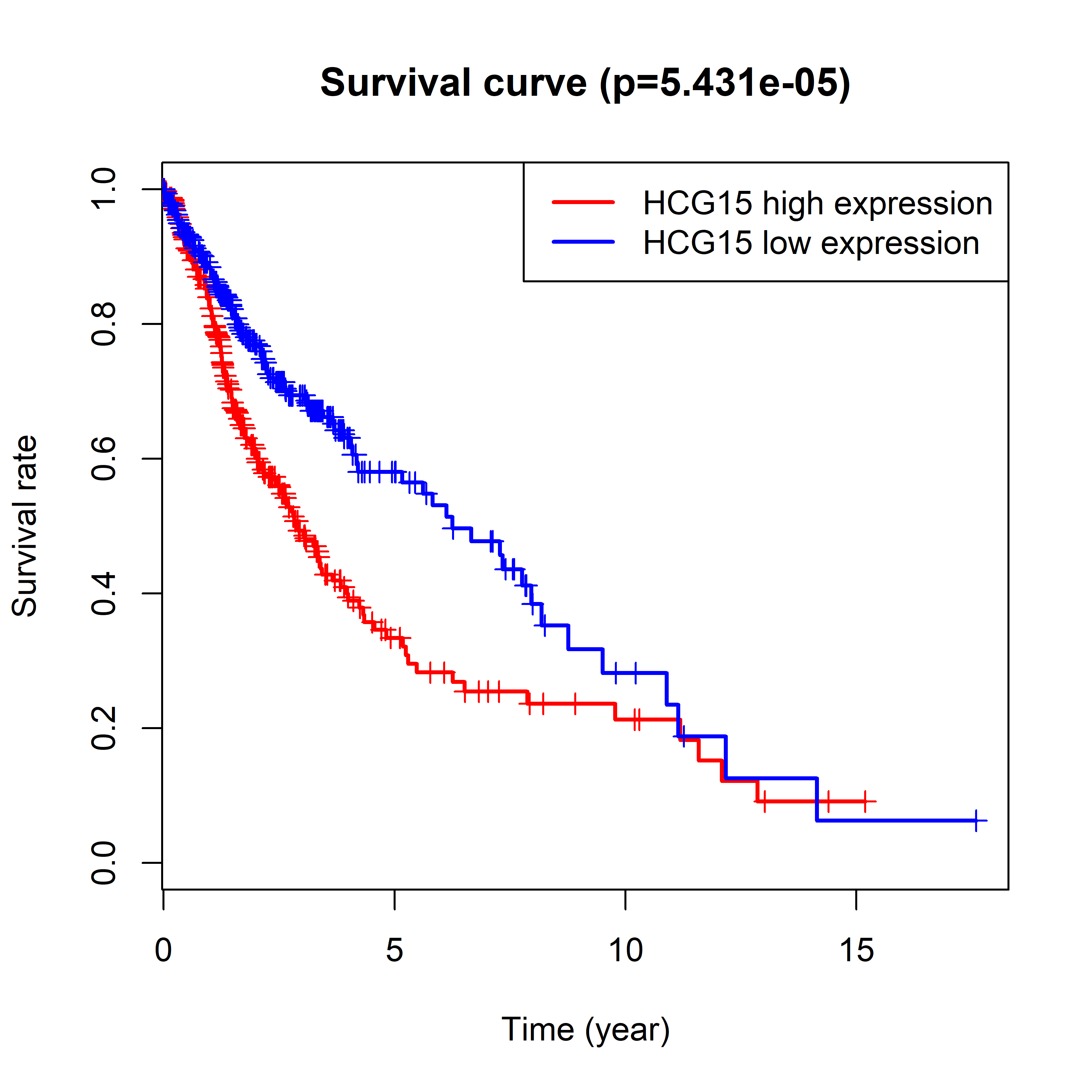

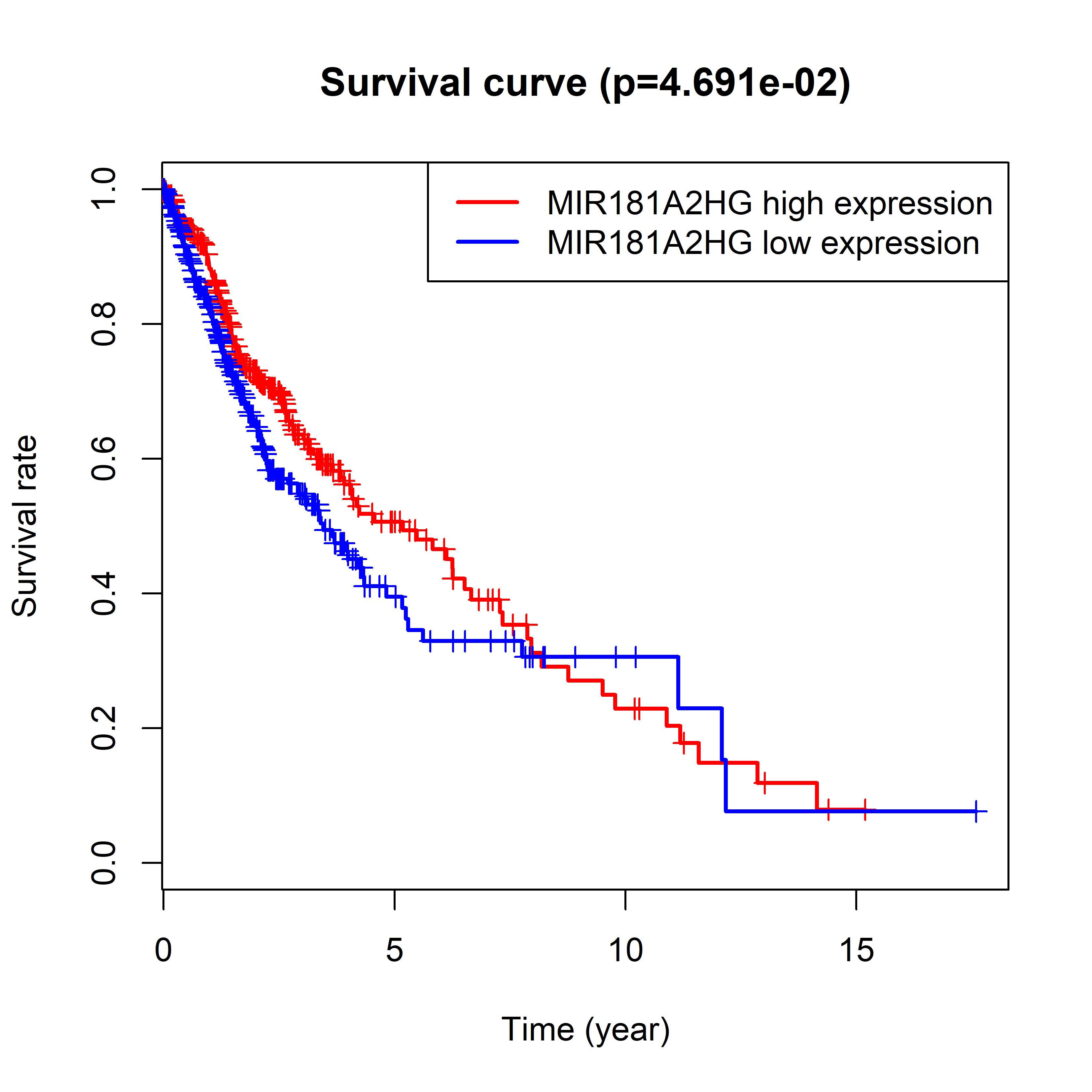

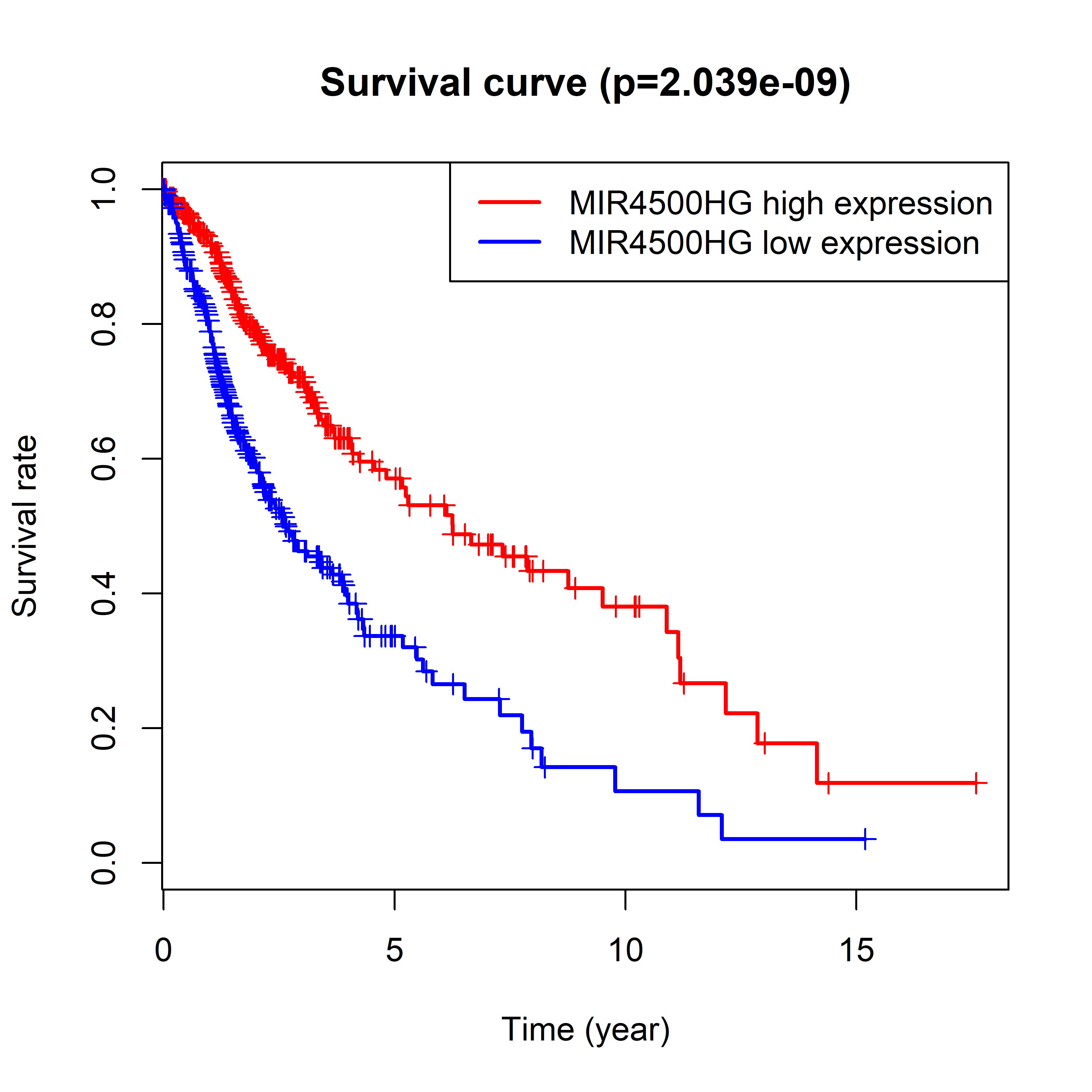

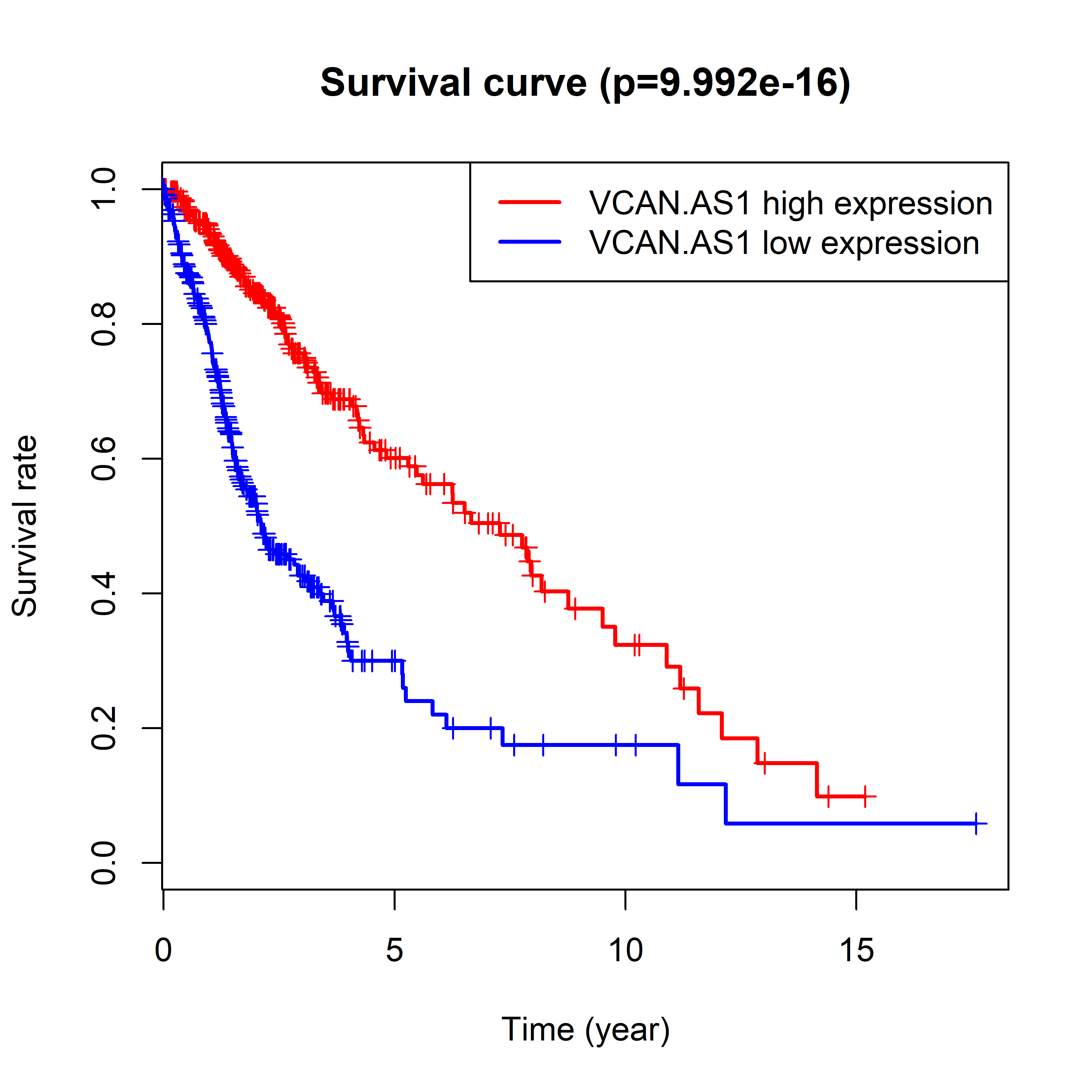

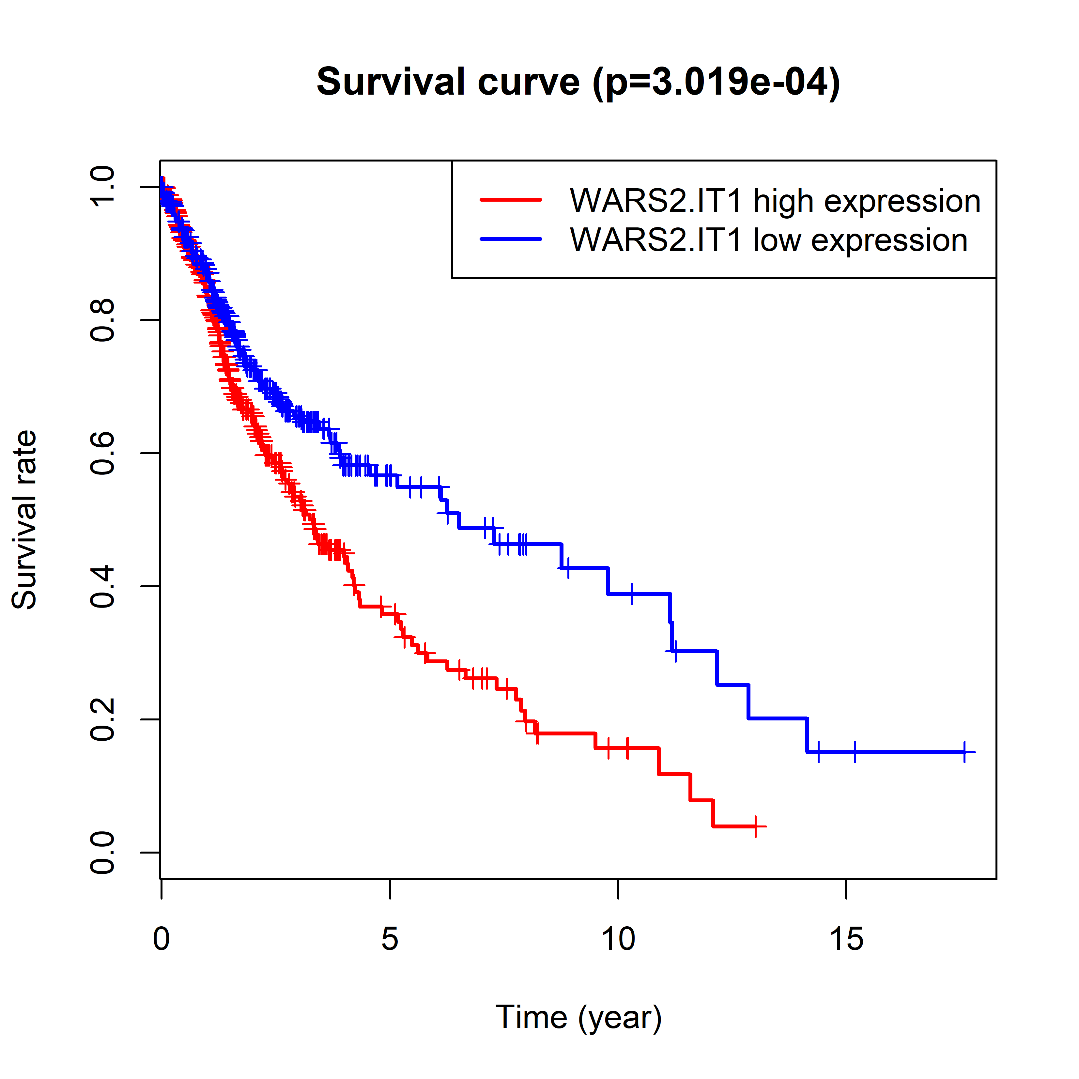

Supplement: S4 File — (DOCX) [file pone.0248634.s008.docx]
